# Comparison of clinical manifestations and immunoreactivities of Meckel's diverticulum with and without gastric heteroplasia

**DOI:** 10.3389/fped.2025.1617214

**Published:** 2025-07-14

**Authors:** Ting Ting Zhang, Xiao Gang Zhou, Peng Cai, Rui Yun Zhang, Zhen Wei Zhu

**Affiliations:** ^1^ Department of Pediatric Surgery, Children’s Hospital of Soochow University, Suzhou, Jiangsu, China; ^2^ Department of Pediatric Surgery, Suzhou Wujiang District Children’s Hospital, Suzhou, Jiangsu, China

**Keywords:** Meckel’s diverticulum, gastric heteroplasia, child, clinical manifestations, immunoreactivities

## Abstract

**Objective:**

This study aimed to characterize the clinicopathological features of pediatric Meckel's diverticulum (MD) and to evaluate the diagnostic performance of imaging modalities, with particular emphasis on the role of heterotopic mucosa.

**Methods:**

A retrospective analysis was conducted on 111 surgically confirmed pediatric MD cases from 2017 to 2023 at a tertiary pediatric center. Patients were stratified into heterotopic and non-heterotopic groups based on histopathological findings. Demographic, clinical, imaging, and immunohistochemical data were analyzed using SPSS version 26.0.

**Results:**

The cohort exhibited a male predominance (ratio 4.29:1) and early age of onset, with 28.8% of patients aged ≤3 years. Heterotopic mucosa, predominantly gastric (74.8%), showed a strong correlation with gastrointestinal bleeding (*p* < 0.0001), whereas non-heterotopic patients more frequently presented with obstruction. Technetium-99m pertechnetate scintigraphy demonstrated significantly higher sensitivity compared with ultrasound (*p* < 0.001). Immunohistochemistry revealed SOX2/MUC5AC expression in heterotopic tissue, in contrast to CDX2 dominance in non-heterotopic patients. All patients underwent successful resection, with no significant differences in outcomes based on the surgical approach employed.

**Conclusions:**

Heterotopic mucosa defines distinct MD subtypes, each with characteristic clinical presentations. Despite its specificity limitations, technetium-99m pertechnetate scintigraphy is optimal for evaluating patients with gastrointestinal bleeding, whereas ultrasound demonstrates poor sensitivity. These findings support the adoption of tailored diagnostic strategies and advocate for universal resection in children with symptomatic MD.

## 1 Introduction

Meckel's diverticulum (MD), often referred to as a diverticulum of the distal ileum, is a specific congenital anomaly. During embryonic development, the vitelline duct typically atrophies and degenerates, starting from the umbilical end and progressing toward the intestinal end. When the umbilical end of the vitelline duct degenerates but the intestinal end remains patent, a blind pouch (Meckel's diverticulum) forms (1–4). The incidence of MD in the general population is approximately 2%, and more than half of affected individuals are infants under the age of 3 (5). MD typically becomes symptomatic only when complications arise. These complications primarily include small intestinal obstruction (30%), acute gastrointestinal bleeding (40%), and acute diverticulitis (20%) (4). Approximately15%–50% of patients have ectopic tissue within the diverticulum (6, 7); however, there are currently no detailed reports available comparing ectopic and non-ectopic tissues in MD. This study retrospectively analyzed the clinical data of 111 children with MD diagnosed by operation and pathology in the Department of Pediatric Surgery, Children's Hospital of Soochow University. Based on pathological examination results, patients were divided into two groups: those with ectopic gastric mucosa and those without. This study aimed to investigate the clinical and pathological features of the two groups, thereby contributing to a better understanding and improved management of MD.

## 2 Methods

### 2.1 Clinical samples

The inclusion criteria included (1) pathologically confirmed Meckel's diverticulum after surgery and (2) complete clinical and imaging data.

The exclusion criteria included (1) pathological examination after surgery ruled out Meckel's diverticulum, (2) severe bleeding and necrosis of the lesion making pathological diagnosis impossible, (3) incomplete clinical data, and (4) the presence of other infectious conditions such as appendicitis or peritonitis.

According to these **criteria**, 116 patients diagnosed with MD in the Department of Pediatric Surgery at Children's Hospital of Soochow University between 2017 and 2023 were recruited. All patients were confirmed through surgical findings and/or histopathological examination. Five patients were excluded because severe hemorrhagic necrosis in their surgical specimens hindered the pathological identification of ectopic mucosal tissue. Consequently, 111 patients formed the final study cohort.

The evaluated parameters included demographic characteristics, morphological and histological assessments of the MD, surgical approaches, clinical presentations, and laboratory results at the time of admission. The study protocol was reviewed and approved by the Ethics Committee of Soochow University Affiliated Children's Hospital (Approval No. 2023CS163).

### 2.2 IHC and quantification

The tissue sections (4 μm) subjected to immunohistochemical (IHC) staining were fixed in freshly prepared 3% H_2_O_2_ with 0.1% sodium azide to quench endogenous peroxidase and then treated with antigen retrieval solution for 15 min. After being placed in blocking reagent for 15 min, the sections were incubated in primary monoclonal antibody [CDX2 (1:200, Proteintech), SOX2 (1:200, Proteintech), MUC5AC (1:200, Proteintech), CK7 (1:200, Proteintech), CK20 (1:200, Proteintech)], overnight at 4°C, followed by incubation with the secondary antibody and ExtrAvidin-conjugated horseradish peroxidase. After, the sections were washed with running water for 5 min and incubated with PAS stain for 15 min. The staining intensity was scored as follows: 0, no staining; 1, weak staining; and 2, moderate to strong staining. The percentage of positively stained cells was scored as follows: 0, <10%; 1, 10%–50%; and 2, >50%. The final score was calculated as the sum of the intensity and quantity scores. A score of >2 indicated positive expression.

### 2.3 Data analysis

An electronic database was created in Excel using data from inpatient records, incorporating double data entry and review for accuracy. Data analysis was performed using SPSS 26.0. Normally distributed data were analyzed with *t*-tests, while enumerated data were assessed using chi-squared tests or Fisher's exact test, with statistical significance set at *p* < 0.05.

## 3 Results

### 3.1 Sex and age distribution

This study included a total of 111 patients with MD, consisting of 90 boys and 21 girls, resulting in a male-to-female ratio of 4.29:1. Among them, 32 patients (28.8%) were ≤3 years old. Ectopic mucosal tissue was identified within the diverticula of 74.8% of the patients, predominantly gastric mucosa. The gastric-to-pancreatic mucosa ratio was 11:1 (Table 1).

**Table 1:** Comparative analysis of ectopic vs. non-ectopic tissues within Meckel's diverticulum, covering demographic, laboratory investigations, clinical presentation, ultrasound findings, and pathological assessment.

Variables	Heterotopic	Non-heterotopic	*P*
Demographic			
Patients (*n*)	83	28	
Gender (male/female)	69/14	21/7	
Age (year)	5.6 ± 3.4	6.6 ± 5.0	0.32
Laboratory			
Hb (g/L)	88.3 ± 25.6	123.5 ± 25.4	<0.0001***
WBC (10^9^/L)	10.1 ± 3.8	14.6 ± 8.1	0.008**
CRP (mg/L)	17.4 ± 40.1	30.5 ± 37.3	0.04*
Etiology			
Intestinal bleeding	62	5	<0.0001***
Intestinal obstruction	16	15	
Diverticulitis	4	6	
Asymptomatic case	1	2	
MD morphometry			
MD length (cm)	3.3 ± 1.4	2.8 ± 1.7	0.09
MD base width (cm)	1.5 ± 0.6	1.4 ± 0.7	0.39
distance of MD to the ileocecal	51.3 ± 18.8	50.7 ± 17.0	0.89
Imaging studies			
^99m^Tc-pertechnetate	50	1	
Ultrasound	21	1	

Hb, hemoglobin; WBC, white blood cell count; CRP, C-reactive protein.

*p < 0.05.

**p < 0.01.

***p < 0.0001.

### 3.2 Clinical manifestations

The 111 pathologically confirmed patients were categorized into two groups: the heterotopic group (*n* = 83) and the non-heterotopic group (*n* = 28). In the heterotopic group, 74.8% of patients exhibited isolated gastric heterotopia, with an overall gastric-to-pancreatic tissue ratio of 11:1. Comparative analysis revealed statistically significant differences between the groups. Specifically, the heterotopic group demonstrated significantly lower hemoglobin (Hb) levels. (*p* < 0.0001). In contrast, the non-heterotopic group exhibited higher white blood cell (WBC) counts (*p* < 0.01) and C-reactive protein (CRP) levels (*p* < 0.05). The clinical manifestations of MD were classified into three primary categories: gastrointestinal bleeding, intestinal obstruction, and diverticulitis. Additionally, some patients were incidentally identified and underwent resection during unrelated abdominal surgeries. Notably, gastrointestinal bleeding occurred in 74.7% of patients in the heterotopic group, compared with only 17.9% in the non-heterotopic group, where intestinal obstruction was the primary presentation in 53.6%. These findings strongly suggest an association between heterotopic mucosa and gastrointestinal bleeding (*p* < 0.0001).

Among the 53 patients with histologically confirmed ectopic mucosa, technetium-99m pertechnetate scintigraphy (^99m^Tc-pertechnetate) correctly identified 50 patients, resulting in a sensitivity of 94.3% [95% confidence interval (CI): 84.1–98.5%]. In the non-heterotopic group, one patient showed a false-positive scan (no ectopic mucosa on pathology), resulting in a specificity of 50.0% (95% CI: 1.3–98.7%). The positive predictive value (PPV) was 98.0%, while the negative predictive value (NPV) was 25.0%. Abdominal ultrasound examinations had a sensitivity of 29.6% (95% CI: 19.1–41.9%) for detecting MD. Specificity could not be calculated because this study lacked a control group without MD. In the heterotopic group, ^99m^Tc-pertechnetate showed significantly higher detection rates compared with ultrasonography (94.3% vs. 29.6%, *p* < 0.001).

All patients underwent surgical treatment. The surgical approaches were generally categorized into two types: one involved laparoscopic exploration followed by an extended umbilical incision to exteriorize the diverticulum for resection. The other approach was direct laparotomy, used to locate and remove the diverticulum, typically performing either a wedge resection of the diverticulum itself or resection of the diverticulum along with adjacent bowel segments. There was no significant difference in patient outcomes between these two surgical methods. Additionally, no significant differences were observed in diverticulum length, diameter, or distance from the ileocecal valve when comparing the heterotopic and non-heterotopic groups (Table 1).

### 3.3 Pathological results

**Figure 1:**
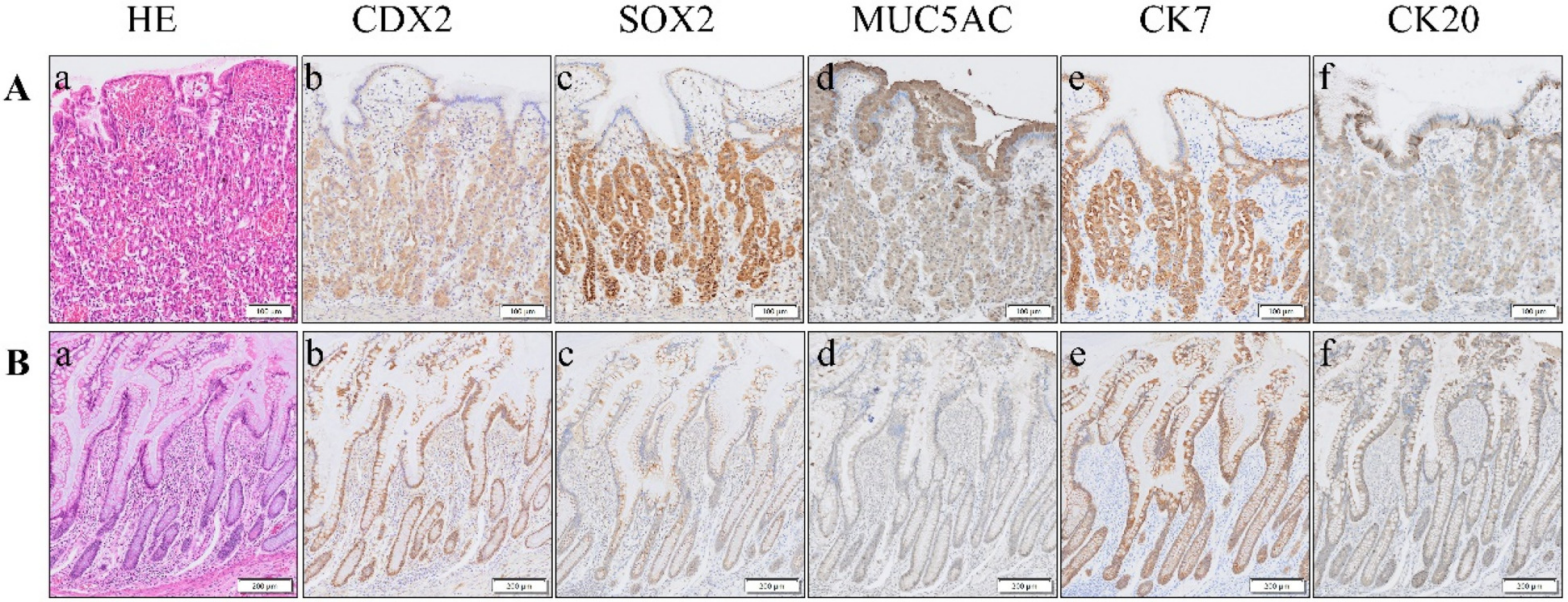
**(A)** Representative case of MD with heterotopic gastric tissue, with ectopic gastric mucosa **(a)**. Immunohistochemistry reveals robust positive staining for SOX2 **(c)**, MUC5AC **(d)**, and CK7 **(e)** within this tissue. In contrast, CDX2 **(b)** and CK20 **(f)** exhibit weak positive expression. **(B)** Representative case of MD lacking ectopic tissue, which is characterized by intestinal epithelial tissue **(a)**. Immunohistochemistry showed that the expressions of CDX2 **(b)** and CK7 **(e)** were strongly positive, SOX2 **(c)** and CK20 **(f)** were weakly positive, and MUC5AC **(d)** was negative. HE, hematoxylin–eosin staining; CDX2, caudal-type homeobox transcription factor 2; SOX2, SRY (sex-determining region Y)-box 2; MUC5AC, recombinant mucin 5 subtype AC; CK7, cytokeratin 7; CK20, cytokeratin 20.

Immunohistochemistry was used to explore differences between MD specimens with and without gastric heterotopia. In those with heterotopic gastric tissue, well-developed gastric-type elements were observed, characterized by a rich presence of chief and parietal cells. These cells exhibited CDX2, SOX2, and MUC5AC expression in the neck mucous cells, alongside distinct CK7 and CK20 patterns (Figure 1A). In patients without heterotopia, the intestinal epithelium was differentiated, showing dominant expression of CDX2 and CK7, but minimal expression of SOX2 and CK20, and no expression of MUC5AC (Figure 1B).

## 4 Discussion

MD is one of the most common congenital malformations of the gastrointestinal tract, resulting from the partial or complete failure of the vitelline duct to degenerate during the early embryonic stage. In 1809, Meckel provided a detailed embryological and clinical description of this congenital condition (8). During embryonic development, the midgut is connected to the yolk sac via the vitelline duct. Toward the end of the fifth week of embryonic development, the lumen of the vitelline duct begins to constrict and gradually close, subsequently atrophying into a fibrous strand that is typically absorbed. However, if this degeneration is incomplete, it can lead to various anatomical abnormalities. The most common form is a diverticulum without any additional attachment, which constitutes MD (4). Clinically, the condition is often first diagnosed following the development of complications such as hematochezia, intestinal obstruction due to compression by the fibrous cord, or diverticulum infection. The specific clinical manifestations of MD and its associated complications are complex and non-specific, making early diagnosis difficult and often leading to missed or misdiagnosed cases. While surgery can effectively cure the vast majority of MD patients who present with clinical symptoms, it has also been reported that the mortality rate associated with MD ranges from 0.4% to 1.5% (9). Therefore, enhancing the clinical understanding of MD and its complications in children holds significant clinical value, facilitating earlier diagnosis and treatment.

Previous studies have found that the male-to-female incidence ratio for asymptomatic MD ranges from 1.5:1 to 4:1 (1). This study observed a significant male predominance, particularly among patients presenting with bleeding symptoms. The male-to-female ratio increased from 3:1 in the non-heterotopic group to 4.9:1 in the heterotopic group. Endogenous hormonal differences, including elevated gastrin levels and increased gastric acid production in male patients, likely contribute to their heightened clinical susceptibility when heterotopic gastric mucosa is present. This may account for the significant male-to-female ratio observed in symptomatic presentations (10).

The most common clinical presentation of symptomatic MD is gastrointestinal bleeding. This typically results from the chronic secretion of digestive enzymes by the ectopic mucosa within the MD, leading to erosion of the diverticulum's mucosa and submucosal vessels, as well as those of the adjacent intestinal wall (5). Studies have found that the prevalence of ectopic mucosal tissue in MD ranges from 15% to 50% (11). Among these ectopic tissues, gastric mucosa is the most common, found in 4.6%–71.0% of cases, followed by pancreatic tissue (0%–12%), and a combination of gastric and pancreatic mucosa occurs in 5% of cases. Furthermore, involvement of other mucosal tissues, such as jejunal, duodenal, colonic, rectal, and endometrial mucosa, is rare (1, 12). Malignant tumors arising in MD are relatively rare, with an incidence rate of only 0.5% to 3.2% (13). Adenocarcinomas associated with MD may originate from various types of heterotopic tissue, including pancreatic tissue, gastric mucosa, or jejunal tissue (14).

During early embryonic gut development, the two transcription factors SOX2 and CDX2 function in distinct regions and are not coexpressed within the same cells. SOX2 is primarily expressed in the more anterior parts of the gut, including the esophagus and stomach (proximal gut), whereas CDX2 is mainly expressed in the more posterior sections, such as the small and large intestines (distal gut). This mutually exclusive pattern of expression helps ensure the proper differentiation and development of the distinct regions of the gut (15). Cytokeratin (CK) is one of the primary proteins composing the epithelial cell cytoskeleton. It plays a crucial role in maintaining cellular structural integrity and morphology, as well as participating in cell movement and differentiation. These proteins are expressed in various types of epithelial cells. Compared to CK7, CK20 is more specifically expressed in gastrointestinal epithelial cells, particularly those lining the colon. However, this study found no significant difference in CK7 and CK20 expression. The heterotopic group exhibited a gastric mucosal phenotype, characterized by SOX2 and MUC5AC expression along with weak CDX2 staining, which is consistent with aberrant foregut differentiation during embryonic development. In contrast, the non-heterotopic patients maintained an ileal signature characterized by strong CDX2 and CK7 expression. These findings provide a molecular basis for the observed clinical differences: the gastric phenotype of heterotopic patients explains their predisposition to acid peptic complications such as bleeding, whereas the native ileal phenotype of the non-heterotopic patients suggests their symptoms likely result from mechanical factors. The SOX2/MUC5AC signature serves as a reliable marker for identifying ectopic gastric mucosa, thereby potentially guiding intraoperative decision-making. By examining the expression patterns of these transcription factors, it is possible to some extent to determine the presence of ectopic tissue within MD, and they can also serve as therapeutic targets for adenocarcinomas associated with MD.

**Figure 2:**
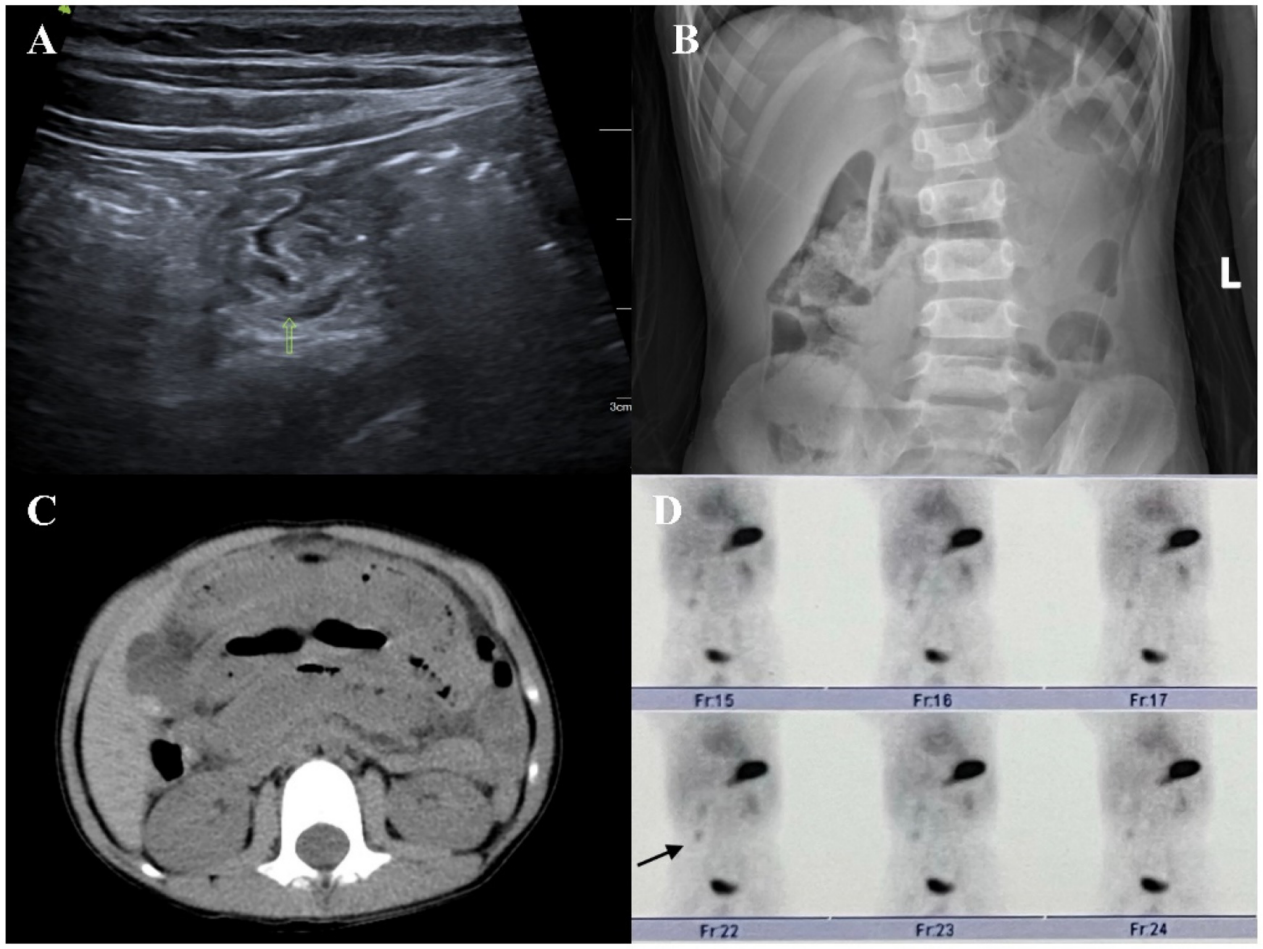
**(A)** On an abdominal ultrasound image, an abnormal intestinal tract can be seen in the right abdomen, where one end is connected with the intestinal tract and the other end is a blind end. A small amount of liquid dark area can be seen on the wall. **(B)** Abdominal plain film image showing a small amount of liquid and a plane of air in the abdominal intestinal tract. **(C)** Abdominal computed tomography image showing part of the intestinal dilatation and effusion, edema, and thickening of the intestinal wall. **(D)** ^99m^Tc-pertechnetate showing an abnormal radioactive concentrated shadow in the right lower abdomen. Its location and morphology did not change significantly within 1 h.

**Figure 3:**
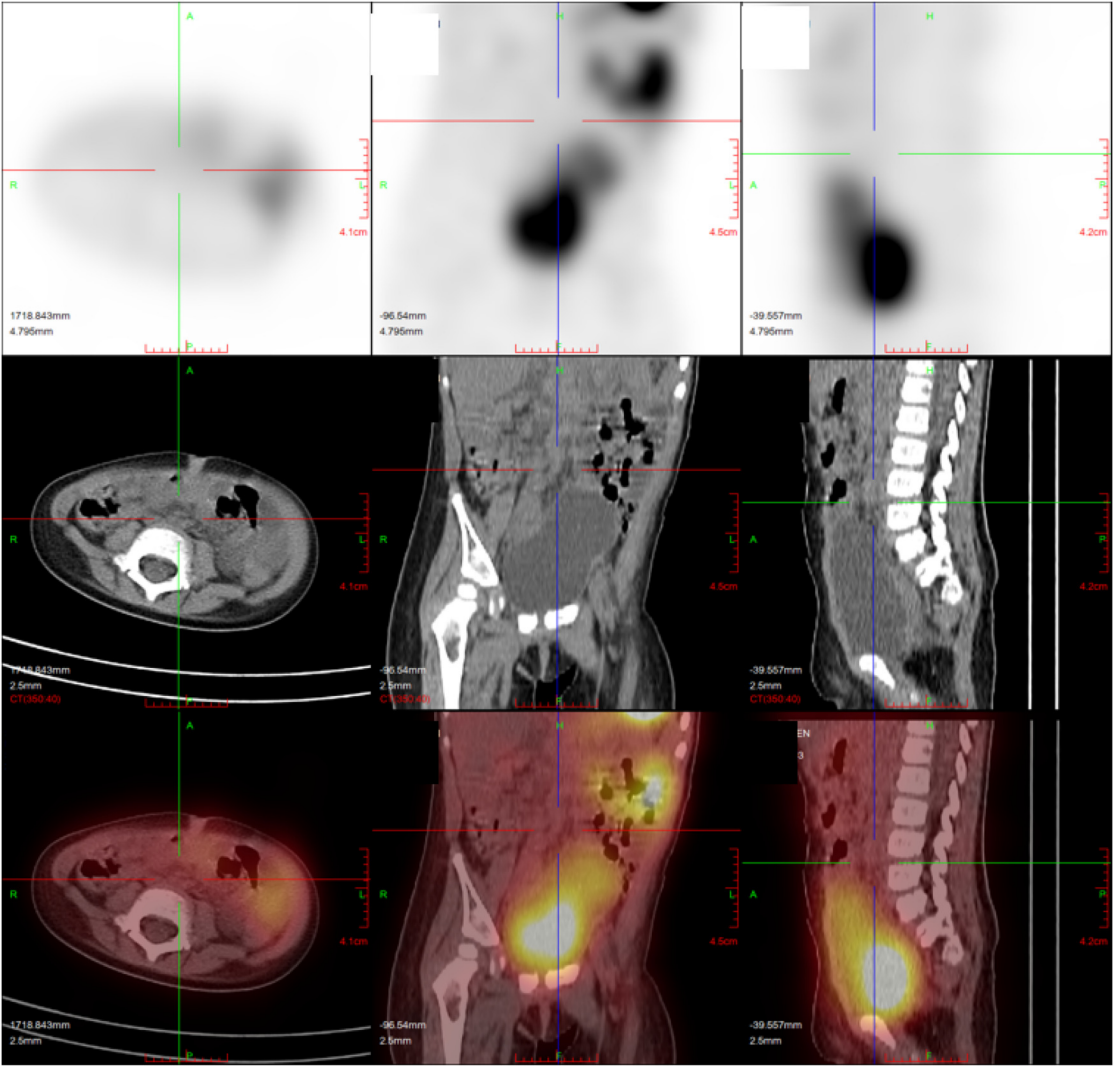
The patient with heterotopic mucosa had false negative findings in ^99m^Tc-pertechnetate (except for a normal radioactive concentrated shadow of bladder and major abdominal vessels, no obvious localized abnormal concentration foci were observed in the abdomen).

In clinical practice, common imaging modalities used for diagnosing MD include abdominal ultrasound, abdominal plain film, abdominal computed tomography, and ^99m^Tc-pertechnetate (Figures 2, 3). Each examination offers distinct advantages. Abdominal ultrasound, commonly used in clinical practice, is effective for visualizing intra-abdominal lesions and their relationship to the surrounding intestines. It often reveals typical features of MD, such as a pouch-like structure located in the right lower abdomen with a distinct three-layered wall pattern (“hyperechoic, hypoechoic, hyperechoic”), thickened mucosa, and absent peristalsis (16). Our study confirms that abdominal ultrasound exhibits low sensitivity for MD (29.6%), meaning that it misses >70% of patients, which limits its role as a standalone diagnostic test. Abdominal plain film and abdominal computed tomography have low sensitivity and low diagnostic value for MD; they can only detect complications such as intestinal obstruction and intestinal perforation caused by MD. Clinically, ^99m^Tc-pertechnetate is considered the most dependable imaging technique for MD. It leverages the rapid uptake of the isotope by both ectopic and normal gastric mucosa, creating positive radioactivity foci (hot spots) that indicate the presence of ectopic tissue. This technique provides diagnostic information and simultaneously enables the precise localization of lesions (17). Our study demonstrates a high sensitivity of 94.3%, aligning with prior reports [89.6% (1)], thereby reinforcing its value in identifying ectopic mucosa, particularly in patients with gastrointestinal bleeding. However, the observed specificity of 50% contrasts sharply with values reported in the literature [97.1% (1)]. This is likely reflecting limitations within our non-heterotopic group (*n* = 2), rather than indicating true diagnostic performance. Therefore, while this scan serves as an excellent initial screening method due to its ability to efficiently identify potential patients, it should not be used alone for definitive diagnosis. For an accurate diagnosis, further evaluation is recommended, potentially involving additional imaging studies or laboratory tests, to complement the findings from the ^99m^Tc-pertechnetate scan and minimize the risk of misdiagnosis.

Currently, it is widely accepted that symptomatic MD should undergo surgical treatment as soon as possible. However, the question of whether asymptomatic MD should be surgically treated remains controversial. A previous study found that the prevalence of MD at autopsy was approximately 1.2%, and its associated mortality rate was approximately 0.001%. The incidence of complications is even higher after the resection of asymptomatic MD compared with leaving it untreated (18). Long-term follow-up indicates that, without intervention for asymptomatic MD, the risk of late complications is extremely low. In our department, transabdominal surgery often includes exploration of the small intestine, particularly the segment between the Treitz ligament and the ileocecal junction. This routine exploration led to the incidental discovery of MD in three children undergoing abdominal surgery for other reasons. Notably, these MDs showed no signs of inflammation or hemorrhage during the operation, yet all were empirically resected. The decision to consider resection was based on the following factors: (1) The World Health Organization and relevant scholars have found that the risk of symptomatic MD does not decrease with age. Consequently, prophylactic resection is generally recommended, especially to prevent serious systemic complications (19). (2) Patients with MD may present with a range of clinical symptoms, including gastrointestinal bleeding, intestinal obstruction, and abdominal infection, among others. In severe cases, MD can even lead to disseminated intravascular coagulation (DIC), shock, and potentially death. (3) According to previous statistical data, the incidence of complications such as incision infection, residual abdominal infection, and intestinal obstruction following MD surgery in our center (3.84%) was not higher than that reported in other studies involving intestinal resection and anastomosis (5.30%). This conclusion is consistent with findings from other research institutions (20). (4) Previous studies have found that the risk of canceration of MD is approximately 70 times higher than in any other part of the ileum, and the median age at diagnosis is 60 years old, which is why prophylactic resection is advocated (21). (5) Children are expected to live significantly longer than adults or the elderly. Therefore, prophylactic resection can effectively prevent various complications from occurring.

In conclusion, managing MD in children presents challenges due to its lack of specific clinical symptoms and typical abdominal signs. Furthermore, the disease tends to progress rapidly once established, increasing the risk of serious complications. For these reasons, we advocate for immediate action: upon clinical suspicion of MD, comprehensive treatment—including surgical intervention—should be initiated without delay. The goal is to promptly halt the disease's progression and prevent the onset of severe complications.

## Data Availability

The original contributions presented in the study are included in the article/Supplementary Material; further inquiries can be directed to the corresponding author.

## References

[1] Hansen CC Soreide K. Systematic review of epidemiology, presentation, and management of Meckel’s diverticulum in the 21st century. Medicine. (2018) 97(35):e12154. 10.1097/MD.0000000000012154 30170459 PMC6392637

[2] Clark JK Paz DA Ghahremani GG. Imaging of Meckel’s diverticulum in adults: pictorial essay. Clin Imaging. (2014) 38(5):557–64. 10.1016/j.clinimag.2014.04.020 24998882

[3] Levy AD Hobbs CM. From the archives of the AFIP. Meckel diverticulum: radiologic features with pathologic correlation. Radiographics. (2004) 24(2):565–87. 10.1148/rg.242035187 15026601

[4] Sagar J Kumar V Shah DK. Meckel’s diverticulum: a systematic review. J R Soc Med. (2006) 99(10):501–5. 10.1177/014107680609901011 17021300 PMC1592061

[5] McDonald JS Horst KK Thacker PG Thomas KB Klinkner DB Kolbe AB. Meckel diverticulum in the pediatric population: patient presentation and performance of imaging in prospective diagnosis. Clin Imaging. (2022) 91:37–44. 10.1016/j.clinimag.2022.07.008 35986976

[6] Koc ZP Ozcan PP Tuncel F Isbir C Usta Y. SPECT/CT in the diagnosis of ectopic gastric mucosa-Meckel’s diverticulum. World J Nucl Med. (2024) 23(3):176–9. 10.1055/s-0044-1787719 39170839 PMC11335389

[7] An J Zabbo CP. Meckel diverticulum. In: StatPearls *[Internet]*. Treasure Island (FL): StatPearls Publishing (2025). 29763135

[8] Kotha VK Khandelwal A Saboo SS Shanbhogue AK Virmani V Marginean EC Radiologist’s perspective for the Meckel’s diverticulum and its complications. Br J Radiol. (2014) 87(1037):20130743. 10.1259/bjr.20130743 24611767 PMC4075535

[9] Rattan KN Singh J Dalal P Rattan A. Meckel’s diverticulum in children: our 12-year experience. Afr J Paediatr Surg. (2016) 13(4):170–4. 10.4103/0189-6725.194671 28051045 PMC5154221

[10] Celebi S. Male predominance in Meckel’s diverticulum: a hyperacidity hypotheses. Med Hypotheses. (2017) 104:54–7. 10.1016/j.mehy.2017.05.014 28673591

[11] Blevrakis E Partalis N Seremeti C Sakellaris G. Meckel’s diverticulum in paediatric practice on Crete (Greece): a 10-year review. Afr J Paediatr Surg. (2011) 8(3):279–82. 10.4103/0189-6725.91665 22248889

[12] Xinias I Mavroudi A Fotoulaki M Tsikopoulos G Kalampakas A Imvrios G. Wireless capsule endoscopy detects Meckel’s diverticulum in a child with unexplained intestinal blood loss. Case Rep Gastroenterol. (2012) 6(3):650–9. 10.1159/000343593 23139657 PMC3493004

[13] Kabir SA Raza SA Kabir SI. Malignant neoplasms of Meckel’s diverticulum; an evidence based review. Ann Med Surg. (2019) 43:75–81. 10.1016/j.amsu.2019.05.017 PMC6582065 31245001

[14] Principe DR Nesper P Metropulos AE Rubin J Marinov MN. Intestinal adenocarcinoma originating from an undiagnosed Meckel’s diverticulum. J Surg Case Rep. (2022) 2022(5):rjac128. 10.1093/jscr/rjac128 35611002 PMC9124546

[15] Raghoebir L Biermann K Buscop-van Kempen M Wijnen RM Tibboel D Smits R Disturbed balance between SOX2 and CDX2 in human vitelline duct anomalies and intestinal duplications. Virchows Arch. (2013) 462(5):515–22. 10.1007/s00428-013-1405-5 23568430

[16] Elsayes KM Menias CO Harvin HJ Francis IR. Imaging manifestations of Meckel’s diverticulum. AJR Am J Roentgenol. (2007) 189(1):81–8. 10.2214/AJR.06.1257 17579156

[17] Low CS Rao N. Imaging of gastrointestinal bleeding: an update. Semin Nucl Med. (2023) 53(6):766–76. 10.1053/j.semnuclmed.2023.06.002 37451934

[18] Zani A Eaton S Rees CM Pierro A. Incidentally detected Meckel diverticulum: to resect or not to resect? Ann Surg. (2008) 247(2):276–81. 10.1097/SLA.0b013e31815aaaf8 18216533

[19] Cullen JJ Kelly KA Moir CR Hodge DO Zinsmeister AR Melton LJ 3rd. Surgical management of Meckel’s diverticulum. An epidemiologic, population-based study. Ann Surg. (1994) 220(4):564–8; discussion 568–569. 10.1097/00000658-199410000-00014 7944666 PMC1234434

[20] Yagnik VD Garg P Dawka S. Should an incidental Meckel diverticulum be resected? A systematic review. Clin Exp Gastroenterol. (2024) 17:147–55. 10.2147/CEG.S460053 38736719 PMC11088382

[21] Thirunavukarasu P Sathaiah M Sukumar S Bartels CJ Zeh H 3rd Lee KK Meckel’s diverticulum–a high-risk region for malignancy in the ileum. Insights from a population-based epidemiological study and implications in surgical management. Ann Surg. (2011) 253(2):223–30. 10.1097/SLA.0b013e3181ef488d 21135700 PMC4129548

